# Barriers to Health Care among the Elderly in Japan

**DOI:** 10.3390/ijerph7041330

**Published:** 2010-03-26

**Authors:** Chiyoe Murata, Tetsuji Yamada, Chia-Ching Chen, Toshiyuki Ojima, Hiroshi Hirai, Katsunori Kondo

**Affiliations:** 1 Hamamatsu University School of Medicine, Department of Community Health and Preventive Medicine, 1-20-1 Handayama, Higashiku, Hamamatsu, 431-3192, Japan; E-Mail: ojima@hama-med.ac.jp; 2 Rutgers University, Department of Economics and Center for Children and Childhood Studies, Camden, NJ 08102, USA; E-Mail: tyamada@crab.rutgers.edu; 3 New York Medical College, School of Health Sciences & Practice, Department of Epidemiology & Community Health, Valhalla, NY 10595, USA; E-Mail: ChiaChing_Chen@nymc.edu; 4 Nihon Fukushi University, Center for Well-being and Society, 5-22-35 Chiyoda Nakaku Nagoya, Aichi, 460-0012, Japan; E-Mails: k-hirai@n-fukushi.ac.jp (H.H.); kkondo@n-fukushi.ac.jp (K.K.)

**Keywords:** cost burden, socioeconomic status (SES), barriers to health care

## Abstract

Japan is undergoing a set of health care reforms aimed at cutting rising health care costs and increasing the efficiency of health care delivery. This empirical study used a large-scale community survey on 15,302 elderly people 65 years and older (56.0% women) conducted in seven municipalities in 2006, to reveal clear-cut evidence of barriers to necessary care. The reasons for not getting health care is attributed to health care cost for the elderly with lower income, while higher income counterparts reported being busy or having a condition not serious enough to seek care.

## 1. Introduction

### 

In addition to unhealthy behaviors, such as smoking, poor diet, or lack of exercise, recent evidence suggests that socioeconomic status is a key underlying factor that influences health. Various studies consistently found that low socioeconomic status is associated with ill health [1–4]. Socioeconomic status assessed by income, education, or occupation is associated with various health problems, such as depression [1,2], hypertension, and functional status [3]. Mortality rates are also higher among individuals of low socioeconomic status [4]. To tackle such health disparities across different socioeconomic strata, the World Health Organization (WHO) recently published a report entitled “Closing the gap in a generation: health equity through action on the social determinants of health, Final Report of the Commission on Social Determinants of Health” [5]. The report clearly states that such inequalities in health arise out of the environment in which we live; grow up, work, and age. In addition, countries at all levels of income, health and illness follow a social gradient: the lower the socioeconomic position, the worse the health of the population is.

Although socioeconomic status is clearly associated to morbidity and mortality, structural links for such associations are less comprehensible. One such link might be knowledge. For example, education provides knowledge and life skills necessary to gain access to information and resources that promote health. This is partly explained by the fact that unhealthy lifestyles are more prevalent among people of low socioeconomic status [6]. Education also provides more opportunities for higher income that may provide better living conditions, such as nutrition, housing, and schooling. Employment status is another important factor in determining health. Unemployed individuals have worse health than their employed counterparts. Less educated people are more likely to be unemployed. Higher socioeconomic status brings us necessary resources to cope with ill health [4,5,7]. Although the association between income and health is stronger in lower income brackets, such associations are observed among richer populations as well. This is supported by a comparative study of 11 European nations which observed health disparity across socioeconomic status groups even in relatively egalitarian societies, such as Sweden or Norway [8]. Furthermore, redistributive policies play an important role in reducing health disparity. A study by Navarro *et al.* analyzed data over a 50-year period in OECD nations and found that redistributive policies have a salutary effect on infant mortality or life expectancy at birth [9].

In addition to income, education, and occupation, access to health care is a vital determinant of health regardless of the nation’s income level [5]. Yet, the access to care problem has not been fully examined in richer nations. Among studies conducted in such nations, Shi and Stevens affirmed that lower socioeconomic status individuals have poorer access to health care by using the national representative sample in the US [10]. Access to care generally encompasses two dimensions. One is physical access, such as the distance to health care facilities or transportation. Another is financial access, such as cost of care or medication. Various studies demonstrated that when co-insurance increased, those with lower income tended to stop going to doctors. Reimbursement under co-insurance system is based on percent basis. This is different from a co-payment, which is a fixed cash amount paid to the beneficiary per procedure or per day in the hospital [11]. Although such systems may offer financial protection, how such a system is framed may influence population health. A study in Japan demonstrated that when co-insurance increased from 20% to 30%, a significant decrease in physician visits was observed among diabetic patients with no complications [12]. A comparative study on three European nations (*i.e.*, France, Germany, and Spain) detailed that an increase in patient cost sharing reduced the frequency of physician visits, especially among people in lower social classes [13]. Similar results are reported in other nations, such as South Korea or Taiwan [14,15]. These studies also underscored that while increases in patient cost sharing reduced visits to physicians, hospital admissions increased especially among lower income people, suggesting that an increase in out of pocket expenditures might have a negative effect on health through lost opportunities for timely care.

#### Brief description of Japanese National Health Insurance System

Japan has maintained a nationwide social health insurance system built on the German social health insurance model for more than 30 years. This system covers almost the entire population and is financed by premiums paid by insured persons, employers, and government compensation. The relatively low-cost, universal health insurance system is recognized as a major achievement. Japanese citizens receive services from any physician or hospital, with no difference in cost, and physicians are, in principle, free to treat or prescribe as they see fit. Under this system Japan’s low infant mortality rate and high life expectancy at birth are among the best in the world [16]. However, pressures are mounting for health care reform to restrain future medical costs in the face of an increasing aging population. In Japan, the National Health Insurance System reimburses on a percentage basis, and the patient pays co-insurance. As the system currently pays 70% of the medical charges, patients (except for children and the elderly) pay the remaining 30%.

Recently, however, there is an issue which requires attention. As of April, 2008, a new health insurance system was launched for the elderly 75 years and older. With the establishment of this system, the government intended to separate the elderly who need more medical attention and use a higher portion of health care resources compared to younger generations. The government intends to restrain an increase in medical costs among the elderly. This system might pose barriers to necessary care, especially among low income individuals.

In this paper, we discuss the associations between socioeconomic status and access to care by investigating possible barriers to health care, examining the scope of health disparities across different socioeconomic groups, and exploring factors which contribute to their associations among the elderly in Japan.

## 2. Methods

### 2.1. Study Population

The present analysis is based on the Aichi Gerontological Evaluation Study (AGES) Project data. The AGES Project is an on-going prospective cohort study that started in two municipalities in Aichi Prefecture, Japan, in 1999. This project aims at investigating factors related to the loss of healthy years, such as functional decline or cognitive impairment, among non-institutionalized elderly subjects aged 65 years or older [17–19]. In 2003, the second wave of surveys was conducted on a random sample of functionally independent, community-dwelling elderly subjects (*i.e.*, who were not eligible for public, long-term nursing care) in fifteen municipalities from three prefectures. During 2006 and 2007, the third wave of surveys was conducted in nine municipalities from three prefectures. For the current study, we used data from the third wave, which contains data on access to health care.

Overall response rate of the third wave survey (mailed questionnaire with three versions) was 60.8% and 39,765 elderly subjects completed the questionnaire. Subjects of the current study were 15,302 elderly people (6,737 men and 8,565 women) living in six municipalities from three prefectures who provided complete data on age and sex, and answered the access to health care version of the survey. Mean age was 74.2 years (age range: 65–100). All respondents were literate and understood the Japanese language well. As a general rule, proxy respondents were not permitted. The study protocol and informed consent procedure were approved by the Ethics Committee in Research of Human Subjects at Nihon Fukushi University.

### 2.2. Study Variables

The variable of interest is health care seeking behaviors. For health care seeking behaviors, we asked if the elderly had a regular place to go when they were ill (regular source of health care), if they had a regular dentist (regular source of dental care), if they underwent health check-ups in the past, and if they ever postponed or stopped seeking health care in the past year (delayed care). We also asked the reasons for not seeking care.

For health status, we used self-assessed health status, and diagnosed illnesses under treatment. Self-assessed health was elicited by asking “What is your current health status: excellent, good, fair, or poor?” with answers dichotomized into excellent/good or fair/poor. To evaluate functional status, the survey asked whether they had difficulty or needed someone’s assistance in performing any of the following ADLs (activities of daily living): walking, bathing, and toileting (0 = without difficulty, 1 = can perform the activity with someone’s partial help, 2 = can’t perform the activity without help of others). Those scoring 0 were considered having no difficulty in performing any ADLs. Diagnosed illnesses were ascertained by asking if they were currently receiving treatment for any of the following: cancer, heart disease, stroke, hypertension, diabetes, obesity, hyperlipidemia, osteoporosis, arthritis, trauma, respiratory illness, gastrointestinal illness, liver disease, mental illness, visual/hearing impairment, dysphagia, urinary disease, sleep disorder, and others.

Health behaviors, such as smoking and drinking, were also assessed, since studies to date indicated that low socioeconomic status is associated with unhealthy lifestyles [6]. As the socioeconomic status measure we used annual equivalized income computed by the square root of the number of people in the household [20]. The purpose of such computation was to adjust the income for family size. Income was defined as pre-tax annual household income, including regular salary, pensions, social security, and any form of temporary earnings during the last year. For the analysis, equivalized income in Japanese yen (¥) was divided into tertiles (low: < ¥1.6 million, middle: ¥1.6 million ≤ ¥ < ¥2.5 million, and high: ¥2.5 million ≤). As of December 2009, one US dollar ($) was the equivalent of ¥87.20 Japanese yen.

### 2.3. Statistical Analysis

We investigated univariate associations between income and health care seeking behaviors, health status, and health behaviors using chi-square tests. In addition, age-adjusted associations were explored by general linear modeling. General linear model is a general model that encompasses both analysis of variance and regression [21]. By using this method, we did analysis of covariance with age in years as a covariate. Finally, odds ratios for delayed care were calculated by adjusting for factors associated with delayed care in univariate analyses. All analyses were carried out using SPSS statistical package for Windows version 13.0.

## 3. Results

### 3.1. Socioeconomic Status and Health

For the association between income and health status/behaviors, the mean percent of the top five illnesses under medical treatment reported by respondents were hypertension (38.7%), visual impairment (16.8%), arthritis (16.3%), heart disease (13.2%), and diabetes (11.3%). Table 1 shows that among these, illnesses most frequently found in lower income individuals were hypertension (39.8%), arthritis (19.2%), and visual impairment (17.9%). Overall, low income elderly subjects had more illnesses requiring medical treatment than their higher income counterparts. As for self-assessed health status, the elderly in the low income group were discernibly lower in health status than middle or higher income groups. The higher percentage of elderly people with low income evidently represents higher dependency in activities of daily living (ADLs) than middle and higher income elderly. We did sub-analyses by gender as well (results are available upon request). Men had slightly more illnesses such as cancer, heart disease, diabetes, or stroke, while women had more problems such as visual impairment, ADL difficulty, and mental illnesses. No clear gender difference was observed regarding the number of illnesses or conditions under treatment and self-assessed health.

### 3.2. Socioeconomic Status, Health Care Seeking Behaviors, and Lifestyle

Table 2 displays health care seeking behaviors, which shows that more elderly subjects of lower socioeconomic status never had health check-ups in the past. Although the percentage of those with a regular source of health care did not differ by socioeconomic status, the proportion of those having a regular source of dental care is higher among higher income elderly. When asked if they ever stopped or postponed necessary medical care in the past year (delayed care), more elderly subjects of low socioeconomic status responded affirmatively. During the past year, about 10% (n = 1,536) of total respondents reported that they did not receive the care they needed. Such tendency was the same both among men and women (detailed results available upon request). More men had a regular source of health/dental care compared to women (23.7% and 21.1% *versus* 17.4% and 16.9% among women, respectively). As for lifestyle, fewer women smoked (2.3% *versus* 21.9% for men) or drank heavily (0% *versus* 0.9% for men). No marked differences by gender were observed regarding health check-up or the experience of delayed care in the past year.

### 3.3. Socioeconomic Status and Reasons for Delayed Health Care

Table 3 depicts reasons for the delay in seeking health care. The statistically significant mean percent of reported reasons by all income levels were cost (24.4%), distance (13.5%), transportation (12.6%), busy (11.4%), and condition not serious enough (26.0%). When stratified by income, low income elderly subjects were more likely to report issues about cost, distance, and transportation problems, while higher income counterparts reported being busy and having a condition not serious enough as a reason for delaying health care. Long waiting hours and dislike of doctors were not statistically significant as reasons for delayed health care. The result of the high percentage (34.3%) of delayed health care due to cost by the low income group demonstrates that low income elderly people are more sensitive to the cost of health care, namely financial burden. The elderly with higher income underestimate their own health problems, as shown by 32.1% for the middle income and 36.2% for the high income groups in variable “condition not serious enough”.

### 3.4. Factors Associated with Delayed Health Care by Income

Logistic regression analysis was conducted to examine the relationship between income levels and delayed health care by adjusting for sex, age, marital status, illnesses, self-assessed health status, smoking, drinking, and regular source of health/dental care, as shown in Table 4. All variables entered here are suggested to be associated with health care seeking behaviors [5]. To test if education confounded the association between income and delayed care, we further constructed model 2 by adding education. However, the association of income with delayed care did not change, indicating that income was associated with delayed care independent of educational background. Low income elderly were 1.41 times more likely to experience delayed health care compared to their higher income counterparts. This means that about 40% more of low income people stopped seeking care in the past year compared to the highest income people. There were no significant differences by gender regarding delayed care in both models.

## 4. Discussion and Implication

The purpose of this paper is to investigate whether difference in income level among the elderly ages 65 years and older, is related to their delayed health care. By using the 3rd wave data of the Aichi Gerontological Evaluation Study Project, we were able to illustrate specific barriers. The results confirm that elderly subjects in the low income group had poorer health status compared to their higher income counterparts as shown in Table 1. Despite unfavorable health status, low income elderly did not have health check-ups in the past and were more likely to postpone or stop receiving the health care. Although diabetes is more prevalent among the low socioeconomic groups in developed high income nations, we did not find significant differences among our population. One such reason is under-diagnosis of the illness. Since responses about illnesses were elicited by self-report, people who did not get medical care might not know that they had diabetes even they actually did. As for lifestyle such as smoking or heavy drinking, no remarkable difference by income group was observed. One reason might be survival effect. Unhealthy individuals might not live long enough to become older. Another is that unhealthy elderly might already have stopped smoking or heavy drinking.

As for reasons for not getting the care, most significant ones among the low income elderly were cost and the distance/transportation. The data of this study, collected from semi-rural areas where medical facilities are sparsely distributed, indicates that 15.8% of low income elderly subjects reported distance as a reason compared to only 9.2% of higher income counterparts and 13.9% of low income elderly subjects also stated the issue as being transportation relative to 6.9% and 7.2% of middle and higher income elderly. Distance and transportation are major concerns, especially in rural areas where medical facilities are sparse. The elderly of those regions often travel hours to get to a hospital. Studies in the UK and US demonstrated that accessibility to public health services is a vital determinant for the health of population [22,23]. These results are congruent with a study in Nigeria by Onwujekwe, who demonstrated that geographical proximity of services is an important factor that affected the utilization of health services [24]. This is often the case in rural areas both in developing and developed nations.

As Babazono *et al.* reported, after the increase in coinsurance from 20% to 30% in Japan there was a decrease in outpatient visits among diabetic patients with no complications [12]. This is of great concern, since studies indicate that increases in cost sharing reduced physician visits but increased hospitalizations [12,13]. The agency for Health Care Research and Quality in the US states that lower socioeconomic individuals are less likely to receive recommended diabetic services and more likely to be hospitalized for diabetes or its complications [25]. Since hypertension and diabetes often have mild symptoms or no symptoms at all, in the first stage of illness, people tend to stop seeking the care they need when out of pocket expenses increase. Delayed care might lead to severe situations and consequences that require more medical treatment such as cardiovascular illnesses, functional impairments, or even dialysis, unless they are treated while their symptoms are mild.

The results of this study confirm that access to health care is not assured equally to the elderly despite a universal health care system in Japan. Under the pressure of curtailing rising health care cost, the Ministry of Health, Labor and Welfare of Japan decided to reconstruct the current health care system and intend to raise co-insurance. However, this might pose additional barriers to care for the old. Among our sample, when stratified by age group, 35.8% of those aged 65−69 reported cost as reasons for not getting the care compared to 20.1% of those over 70. This is important since they were in different payment scheme at the time of the survey. Those aged 65−69 paid 30% as co-insurance while those over 70 paid only 10%. Such difference in co-insurance might pose higher financial burden among the 65−69 old people. For the implementation of health care reform, evaluating the impact of increases in co-insurance or premiums, especially on low income people, might be necessary.

Finally, we need to touch upon some limitations. We used data on older people. This might lead to an underestimation of the association between income and delayed care. Since most of our samples already live on pensions, the quality of health care they receive might be quite similar since they are under the same insurance system. To truly assess the impact of socioeconomic status on health care seeking behaviors, we may need younger generations since we expect such inequalities in health might even greater among younger generations. In addition, dislike of doctors or condition not serious enough also emerged as reasons for not getting health care, irrespective of their income level. Financial burden might not be the sole reason for the delayed care. It is imperative to investigate possible barriers to health care not only from behavioral but from societal perspective as well.

## 5. Conclusion

Even in Japan, with its universal health care system, low income elderly were more likely to postpone or stop seeking health care in the past year, indicating a health disparity across socioeconomic status. Most reasons reported by the low income elderly were the burden of health care costs and distance. As causes of differential health-care access, there is a lack of available services and economic barriers to care [5]. The WHO urged for action by stating that “National governments ensure public sector leadership in health-care systems financing, focusing on tax-/insurance-based funding, ensuring universal coverage of health care regardless of ability to pay, and minimizing out of-pocket health spending” [5]. Providing financially accessible services for everyone irrespective of their income as well as creating basic health care facilities in remote areas might be important since delayed health care may lead to worse health consequences.

A change of regime occurred in Japan in August 2009, and the new government is facing challenges of reviewing the health care system for the elderly. Further study requires investigating the consequences of delayed care by using follow-up data for better health care policies.

## Figures and Tables

**Table 1.** Association between health status and income (N = 15,302).

	Income Tertile			
Health Status	Low Income (%)	Middle Income (%)	High Income (%)	P values
Self-assessed health status (fair/poor)	34.6	26.2	22.1	<0.001
Functional status (ADL dependence)	5.1	3.1	3.2	<0.001
Diagnosed illnesses under treatment	78.2	75.6	75.0	<0.010
Heart disease	12.9	12.9	13.0	0.994
Stroke	2.7	2.2	1.8	<0.050
Cancer	3.5	4.0	3.9	0.449
Hypertension	39.8	38.6	36.3	<0.010
Obesity	4.9	3.8	3.2	<0.010
Diabetes (Type 1&2)	11.8	11.2	11.9	0.599
Gastrointestinal illness	9.2	7.4	6.9	<0.010
Respiratory illness	3.6	3.7	3.2	0.439
Liver disease	2.9	2.8	2.5	0.503
Arthritis/neuralgia	19.2	13.9	13.9	<0.001
Osteoporosis	8.3	5.3	5.8	<0.001
Visual impairment	17.9	15.2	15.3	<0.010
Hearing impairment	9.6	6.3	5.5	<0.001
Urinary illness	11.5	8.3	7.3	<0.001
Sleep disorder	9.9	7.0	6.1	<0.001
Mental illness	1.7	1.1	0.5	<0.001
Others	6.2	8.6	9.3	<0.001

All figures in the table are percentages (not adjusted).

p < 0.001, p < 0.01, and p < 0.05 represent p values for statistically significant level of 95% for the two-tailed test. N stands for the number of observations.

**Table 2.** Association between health care seeking behaviors, lifestyle, and income by age-adjusted % (N = 15,302).

	Income tertile			
Health care seeking behaviors	Low Income (%)	Middle Income (%)	High Income (%)	P values
Regular source of health care (no)	20.6	22.2	20.1	0.760
Regular source of dental care (no)	22.0	17.2	16.4	0.063
Health check-up (never)	27.5	20.7	17.4	0.159
Delayed care (yes)	12.0	9.1	8.3	<0.050
Health behaviors				
Current smoking	12.4	11.8	11.6	0.524
Everyday drinking (3 Go^a^) ≈ 540cc	0.8	0.4	0.4	0.335

All figures in the table are percentages adjusted for mean age using general linear model.

a: “Go” is a Japanese unit of measurement in which one Go is equivalent of 20g ethanol (pure alcohol).

p < 0.05 represents statistically significant level of 95% for the two-tailed test. N stands for the number of observations.

**Table 3.** Association between reasons for delayed health care and income (N = 1,536).

	Income tertile			
Reasons for delayed health care	Low Income (%)	Middle Income (%)	High Income (%)	P values
Long waiting hours	30.3	31.5	27.6	0.550
Cost	34.3	22.5	13.8	<0.001
Distance	15.8	11.3	9.2	<0.050
Don’t know where to go	6.7	4.6	3.8	0.170
Transportation problem	13.9	6.9	7.2	<0.010
Dislike doctors	24.5	23.7	23.4	0.927
Busy	8.4	12.8	13.1	0.061
Condition not serious enough	17.3	32.1	36.2	<0.001
Others	3.3	5.1	6.2	0.150

All figures in the table are percentages (not adjusted).

p < 0.001, p < 0.01, and p < 0.05 represent p values for statistically significant level of 95% for the two-tailed test. N stands for the number of observations.

**Table 4.** Odds ratios for delayed care (N = 15,302).

	Low Income	Middle Income	High Income
Model 1	1.37 (1.16–1.61)	1.05 (0.89–1.23)	1.00
	p < 0.001	p = 0.937	---
Model 2	1.41 (1.20–1.67)	1.07 (0.91–1.26)	1.00
	p < 0.001	p = 0.443	---

Model 1 was adjusted for sex, age, marital status, illnesses, self-assessed health status, smoking, drinking, and regular source of health/dental care.

Model 2 was further adjusted for education in addition to variables entered in the model 1.

P values are calculated with 95% confidence interval for logistic regression analyses.

High income is the reference category in logistic regression models.

N stands for the number of observations. The full results are available upon request.

## References

[1] Weissman MM Bland RC Canino GJ Faravelli C Greenwald S Hwu HG Joyce PR Karam EG Lee CK Lellouch J Lepine JP Newman SC Rubio-Stipec M Wells JE Wickramaratne PJ Wittchen H Yeh EK Cross-national epidemiology of major depression and bipolar disorder JAMA 1996 276 293 299 8656541

[2] Lorant V Deliège D Eaton W Robert A Philippot P Ansseau M Socioeconomic inequalities in depression: a meta-analysis Am. J. Epidemiol 2003 157 98 112 12522017 10.1093/aje/kwf182

[3] Beydoun MA Popkin BM The impact of socio-economic factors on functional status decline among community-dwelling older adults in China Soc. Sci. Med 2005 60 2045 2057 15743653 10.1016/j.socscimed.2004.08.063

[4] Kagamimori S Gaina A Nasermoaddeli A Socioeconomic status and health in the Japanese population Soc. Sci. Med 2009 68 2152 2160 19375838 10.1016/j.socscimed.2009.03.030

[5] Commission on Social Determinants of Health (CSDH) Closing the Gap in a Generation: Health Equity through Action on the Social Determinants of Health Final Report of the Commission on Social Determinants of Health World Health Organization Geneva, Switzerland 2008 94 106

[6] Fukuda Y Nakamura K Takano T Socioeconomic pattern of smoking in Japan: income inequality and gender and age differences Ann. Epidemiol 2005 15 365 372 15840550 10.1016/j.annepidem.2004.09.003

[7] Marmot M Status Syndrome1st ed Bloomsbury Publishing London, UK 2004 83 105

[8] Cavelaars AE Kunst AE Geurts JJ Crialesi R Grötvedt L Helmert U Lahelma E Lundberg O Matheson J Mielck A Mizrahi A Rasmussen NK Regidor E Spuhler T Mackenbach JP Differences in self reported morbidity by educational level: a comparison of 11 western European countries J. Epidemiol. Commun. Health 1998 52 219 227 10.1136/jech.52.4.219 PMC1756698 9616407

[9] Navarro V Muntaner C Borrell C Benach J Quiroga A Rodríguez-Sanz M Vergés N Pasarín MI Politics and health outcomes Lancet 2006 16 1033 1037 16980120 10.1016/S0140-6736(06)69341-0

[10] Shi L Stevens GD Vulnerability and unmet health care needs. The influence of multiple risk factors J. Gen. Internal Med 2005 20 148 154 15836548 10.1111/j.1525-1497.2005.40136.x PMC1490048

[11] Koch AL Financing health services Introduction to Health Services4th ed Williams SJ Torrens PR Delmar Publishers Inc New York, NY, USA 1993 299 331

[12] Babazono A Miyazaki M Imatoh T Une H Yamamoto E Tsuda T Tanaka K Tanihara S Effects of the increase in co-payments from 20 to 30 percent on the compliance rate of patients with hypertension or diabetes mellitus in the employed health insurance system Int. J. Technol. Assessment Health Care 2005 21 228 233 15921063

[13] Lostao L Regidor E Geyer S Aïach P Patient cost sharing and social inequalities in access to health care in three western European countries Soc. Sci. Med 2007 65 367 376 17544192 10.1016/j.socscimed.2007.05.001

[14] Kim J Ko S Yang B The effects of patient cost sharing on ambulatory utilization in South Korea Health Policy 2005 72 293 300 15862637 10.1016/j.healthpol.2004.09.002

[15] Huang JH Tung CM The effects of outpatient co-payment policy on healthcare usage by the elderly in Taiwan Arch. Gerontol. Geriatr 2006 43 101 116 16280181 10.1016/j.archger.2005.09.007

[16] OECD Health Data Statistics and Indicators for 30 Countries Available online: http://www.oecd.org/health/healthdata (accessed on December 2009).

[17] Murata C Kondo K Hirai H Ichida Y Ojima T Association between depression and socio-economic status among community-dwelling elderly in Japan: the Aichi Gerontological Evaluation Study (AGES) Health Place 2008 14 406 414 17913562 10.1016/j.healthplace.2007.08.007

[18] Ichida Y Kondo K Hirai H Hanibuchi T Yoshikawa G Murata C Social capital, income inequality and self-rated health in Chita peninsula, Japan: a multilevel analysis of older people in 25 communities Soc. Sci. Med 2009 69 489 499 19523728 10.1016/j.socscimed.2009.05.006

[19] Aida J Hanibuchi T Nakade M Hirai H Osaka K Kondo K The different effects of vertical social capital and horizontal social capital on dental status: a multilevel analysis Soc. Sci. Med 2009 69 512 518 19573968 10.1016/j.socscimed.2009.06.003

[20] Dalstra JA Kunst AE Mackenbach JP EU Working Group on Socioeconomic Inequalities in Health A comparative appraisal of the relationship of education, income and housing tenure with less than good health among the elderly in Europe Soc. Sci. Med 2006 62 2046 2060 16221515 10.1016/j.socscimed.2005.09.001

[21] Norman GR Streiner DL Variations on Linear Regression PDQ Statistics3rd ed B.C. Decker Inc Hamilton, Canada 2003

[22] Barnett S Roderick P Martin D Diamond I A multilevel analysis of the effects of rurality and social deprivation on premature limiting long term illness J. Epidemiol. Commun. Health 2001 55 44 51 10.1136/jech.55.1.44 PMC1731764 11112950

[23] Yamada T Chen CC Yamada T Chiu IM Smith J Healthcare services accessibility of children in the USA Appl. Econ 2009 41 437 450

[24] Onwujekwe O Inequities in healthcare seeking in the treatment of communicable endemic diseases in Southeast Nigeria Soc. Sci. Med 2005 61 455 463 15893059 10.1016/j.socscimed.2004.11.066

[25] Agency for Healthcare Research and Quality National Healthcare Disparities Report: Inequality in Quality Exists Agency for Healthcare Research and Quality Rockville, MD, USA 2003 Available online: http://www.ahrq.gov/QUAL/nhdr03/nhdrsum03.htm#Inequality (accessed on 7 March 2008).

